# Exploring the Benefits of Molecular Testing for Gonorrhoea Antibiotic Resistance Surveillance in Remote Settings

**DOI:** 10.1371/journal.pone.0133202

**Published:** 2015-07-16

**Authors:** Ben B. Hui, Nathan Ryder, Jiunn-Yih Su, James Ward, Marcus Y. Chen, Basil Donovan, Christopher K. Fairley, Rebecca J. Guy, Monica M. Lahra, Mathew G. Law, David M. Whiley, David G. Regan

**Affiliations:** 1 The Kirby Institute, UNSW Australia, Sydney, New South Wales, Australia; 2 Sexual Health and Blood Borne Virus Unit, Centre for Disease Control, Department of Health, Darwin, Northern Territory, Australia; 3 South Australian Health and Medical Research Institute, North Terrace, Adelaide, South Australia, Australia; 4 Central Clinical School, Monash University, Melbourne, Victoria, Australia; 5 Melbourne Sexual Health Centre, Alfred Health, Carlton, Victoria, Australia; 6 School of Population and Global Health, University of Melbourne, Melbourne, Australia; 7 Sydney Sexual Health Centre, Sydney Hospital, Sydney, New South Wales, Australia; 8 WHO Collaborating Centre for STD, Microbiology Department, South Eastern Area Laboratory Services, Prince of Wales Hospital, Sydney, New South Wales, Australia; 9 Queensland Paediatric Infectious Diseases Laboratory, Queensland Children’s Health Services, Queensland, Australia; 10 Queensland Children’s Medical Research Institute, University of Queensland, Queensland, Australia; Centers for Disease Control and Prevention, UNITED STATES

## Abstract

**Background:**

Surveillance for gonorrhoea antimicrobial resistance (AMR) is compromised by a move away from culture-based testing in favour of more convenient nucleic acid amplification test (NAAT) tests. We assessed the potential benefit of a molecular resistance test in terms of the timeliness of detection of gonorrhoea AMR.

**Methods and Findings:**

An individual-based mathematical model was developed to describe the transmission of gonorrhoea in a remote Indigenous population in Australia. We estimated the impact of the molecular test on the time delay between first importation and the first confirmation that the prevalence of gonorrhoea AMR (resistance proportion) has breached the WHO-recommended 5% threshold (when a change in antibiotic should occur). In the remote setting evaluated in this study, the model predicts that when culture is the only available means of testing for AMR, the breach will only be detected when the actual prevalence of AMR in the population has already reached 8 – 18%, with an associated delay of ~43 – 69 months between first importation and detection. With the addition of a molecular resistance test, the number of samples for which AMR can be determined increases facilitating earlier detection at a lower resistance proportion. For the best case scenario, where AMR can be determined for all diagnostic samples, the alert would be triggered at least 8 months earlier than using culture alone and the resistance proportion will have only slightly exceeded the 5% notification threshold.

**Conclusions:**

Molecular tests have the potential to provide more timely warning of the emergence of gonorrhoea AMR. This in turn will facilitate earlier treatment switching and more targeted treatment, which has the potential to reduce the population impact of gonorrhoea AMR.

## Introduction

The gonorrhoea rate is disproportionately high in some Indigenous populations in remote Australia compared with urban areas, with rates reported to be up to 35 times higher [1, 2]. Prevalence of 7–8% has been reported for 16–34 years old and even higher for 16–19 year olds at more than 10% [3]. Currently, most gonorrhoea infections diagnosed in remote Indigenous communities are sensitive to and treated with penicillin, whereas the predominant gonorrhoea strains circulating in urban Australia and neighbouring countries are resistant to penicillin [4]. It is therefore likely that penicillin-resistant gonorrhoea will eventually be introduced and take hold within remote communities and will compromise the effectiveness of existing control strategies.

Strengthening surveillance for antimicrobial resistance (AMR) in settings where gonorrhoea prevalence is high is a key strategy of the World Health Organisation (WHO) [5] and this is necessarily a bacterial culture-based activity. However 50–90% of the gonorrhoea infections from remote regions are diagnosed using nucleic acid amplification tests (NAAT) [6–9] primarily because of distance and transport considerations as well as the convenience and sensitivity of NAAT-based diagnosis. With the increasing trend toward use of NAAT for diagnosis, the number of samples available for culture is expected to reduce even further, possibly to a level that will not be adequate or sufficiently representative for AMR surveillance [10].

Molecular tests specifically designed to identify genetic mutations that confer resistance have the potential to enhance AMR surveillance by improving coverage and representativeness [11]. For example, a molecular test to detect penicillinase-producing *Neisseria gonorrhoeae* (PPNG) has been recently described [12] and is now in use to enhance the surveillance of penicillin resistance in remote Western Australia where a penicillin-based treatment strategy is in use. Data from the PPNG NAAT-based surveillance is used to inform clinical guidelines for this region [9]. The widespread use of such tests on diagnostic samples could enhance AMR surveillance and enable a more timely response to the emergence of treatment-resistant gonorrhoea. The WHO guidelines for STI management state that a treatment should have a 95% cure rate to be considered effective. [5]. By implication, when the treatment failure rate exceeds 5% with the current treatment, there should be a switch to a new effective treatment. We refer to this henceforth as the 5% threshold.

In this study we describe results from a mathematical model of genital gonorrhoea transmission in a remote community setting that we developed to assess the potential impact of a molecular test on the timeliness of detection of gonorrhoea AMR.

## Methods

An individual-based mathematical model was developed to describe the transmission of gonorrhoea in a remote Indigenous population in Australia. The model is an extension of a previously published model that we used to evaluate the importance of population mobility for gonorrhoea transmission in remote Indigenous communities of Australia [13]. While the model is parameterised using demographic, sexual behaviour and mobility data for this setting, our approach can be applied to other settings.

The modelled population represents sexually active individuals aged 15–35 years residing in one larger centre (comprising 4000 individuals) and four small communities (comprising 250 individuals each) that are “satellites” of the larger centre from a population mobility perspective [13]. Gender- and age-specific mortality rates were adjusted to maintain the gender-age distribution as described in census data [14].The model tracks age, gender, location, gonorrhoea infection status and sexual behaviour on a daily basis, while population mobility is captured based on census data using modelling techniques as previously described [13]. Table 1 lists the demographic and behavioural parameters used in the model. A more detailed description of the model is provided in the S1 Technical Appendix.

**Table 1:** Demographic and behavioural parameters.

Behaviour parameters	Value				Reference
Age distribution	Age	Male		Female	[14]
	15–20	33%		31%	
	20–25	27%		27%	
	25–30	22%		23%	
	30–35	18%		19%	
Number of residents in each location	Regional centre		4000		Assumption, based on the size of the regions listed in [30]
	Remote satellite communities		250		Assumption, based on the size of the regions listed in [30]
Proportion of population away from home at any time	Age	Male		Female	[31]
	15–20	10.0%		11.1%	
	20–25	9.4%		10.1%	
	25–30	9.5%		8.4%	
	30–35	9.2%		7.9%	
Time spent away from home upon each episode of travel	2–14 days				[13]
Probability of seeking additional partners while away from home	5% of population				[13]
Proportion of population that have at least one regular partners in past 6 months	54.8% (a)				[32], adjusted to exclude individuals with no partnerships
Proportion of modelled population that has at least one casual partner in past 6 months (a)	72.4%				[32], adjusted to exclude individuals with no partnerships
Proportion of population with more than 1 casual partner in 6 months	40.0% of (a)				[32]
Proportion of population having a casual and regular partner concurrently	27.6% of (a)				[32]
Duration of regular partnerships	2 years				[32]
Frequency of sexual acts within partnerships	3 per week				Assumption based on data for general Australian population [33]
Average number of sexual partners for individuals aged <30	5				[32]
Condom usage (%)	Regular		20.6		[32]
	Casual		39.2		[32]
Screening coverage	44% population screened per year				[15]
Treatment rate for symptomatically infected individuals who seek treatment	85% treated within 21 days				[15]
Proportion of index cases whose partners are notified and treated	24% per index case				[15]

We assume that initially all gonorrhoea infections in the population are sensitive to treatment but that treatment failure may occur, on rare occasions, for reasons not related to AMR (e.g. adherence failure). Treatment-resistant gonorrhoea is then introduced into the population through periodic importation. We use the model to estimate the impact of the molecular test on the time delay between first importation and the first confirmation that the prevalence of gonorrhoea AMR has breached the 5% threshold. For simplicity, we consider a single generic treatment-sensitive strain and single treatment-resistant strain, for each of which a generic effective treatment exists. We do not consider specific gonorrhoea strains or antibiotics.

We assume that some asymptomatic individuals infected with gonorrhoea in remote Indigenous populations will be tested and treated through general health check-ups and screening, while a proportion of those with symptoms will actively seek treatment. Of those testing positive to gonorrhoea, 85% will receive treatment within 21 days [15] but some will remain infected post-treatment due to treatment failure as a result of AMR or other factors such as non-adherence. We assume that 44% of the population will be tested for STI annually through general health check-ups and screening [15], using a diagnostic test of 100% sensitivity [16].

In contrast to urban Australia, it is thought that a substantial proportion of symptomatically infected individuals in remote Indigenous communities do not seek treatment for a combination of reasons including lack of knowledge about sexually transmissible infections (STIs) and the availability of treatment, anxiety and stigma associated with STIs, and accessibility to appropriate health services. [17, 18]. While evidence for this is anecdotal we assume that only 40% of individuals with symptoms seek and receive treatment, a proportion arrived at through calibration to notification data. Combined with treatment through general check-ups and screening, this rate of symptomatic treatment results in ~2700 treatments per 100 000 population per year, corresponding roughly to the reported gonorrhoea notification rate among 16–30 year-old residents of remote Indigenous communities [1].

### Properties of treatment sensitive and treatment-resistant gonorrhoea

We assume that an endemic prevalence of treatment-sensitive gonorrhoea is already established before the importation of treatment-resistant gonorrhoea. Prior to the first importation, the model is calibrated to yield a constant gonorrhoea prevalence of 7–8% [3].

To date, the identification of treatment-resistant gonorrhoea has been a rare occurrence in remote Australia [4]. In this study, therefore, our assumptions regarding the natural history of treatment-resistant gonorrhoea and responses from health care service providers are necessarily hypothetical. Furthermore, there is no substantive evidence suggesting that the natural history of treatment-resistant strains is different from that of treatment-sensitive strains. We therefore assume that treatment-sensitive and treatment-resistant gonorrhoea strains differ only in their respective treatment failure rates. We assume a failure rate of 100% for treatment-resistant infections. For treatment-sensitive infections, we assume there is a failure rate of 5% for reasons not related to AMR. We make this assumption as a worst-case scenario because reliable data on treatment failure in the remote Indigenous setting are not available but a 95% effective treatment would be considered effective under WHO guidelines [5]. Values assigned to gonorrhoea natural history parameters used in the model apply to both treatment-sensitive and treatment-resistant gonorrhoea and are listed in Table 2.

**Table 2:** Infection parameters for treatment-sensitive and treatment-resistant gonorrhoea.

Infection parameters	Value		Reference
Probability of infection being recognised as symptomatic	Male	0.45	[34]
	Female	0.14	[34]
Average duration of latent period	4 days		Assumption
Average duration of infectious period	185 days		[35–37]
Average duration of immunity following resolution of infection	7 days		Assumption
Transmission probability per unprotected sex act	Male to female	0.50	[38–40]
	Female to male	0.22	[38–40]

**Fig 1:**
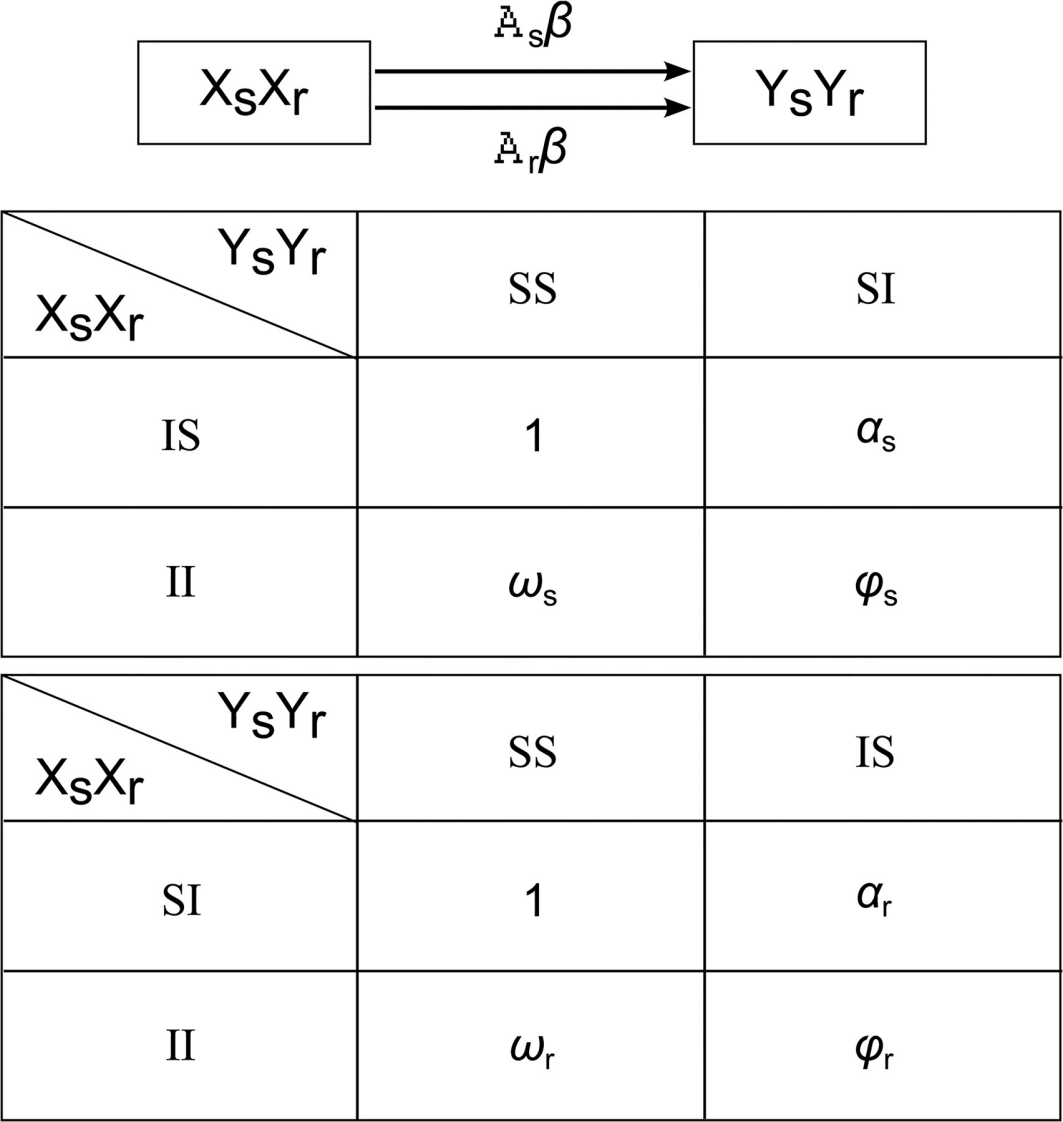
Transmission mechanism for treatment-sensitive and treatment-resistant gonorrhoea as implemented in the model. Xs, Xr denote the infection status of person X: Xs = S if X is susceptible to treatment-sensitive gonorrhoea; Xs = I if X is infected with treatment-sensitive gonorrhoea. Similarly, Xr = S or Xr = I if X is susceptible or infected with treatment-resistant gonorrhoea, respectively. The diagram represents transmission from person X to person Y through unprotected sex act (transmission from Y to X is omitted in this diagram). The parameter *β* is the transmission probability per unprotected sex act. The parameters *A*_***s***_ and *A*_***r***_ are scalar adjustment to the transmission probability for treatment-sensitive and treatment-resistant gonorrhoea, respectively, and their value will be based on the infection status of person X and person Y as defined in the figure. *A*_***s***_ and *A*_***r***_ are equal to zero for all combinations not listed.

In any population in which treatment-sensitive and treatment-resistant strains are circulating, there is a finite probability (the value of which will depend on the prevalence of each strain) that an individual infected with treatment-sensitive gonorrhoea will engage in sexual contact with an individual infected with treatment-resistant gonorrhoea. In our model we assume that in this circumstance, transmission can occur such that one or both individuals can become co-infected with both strains [19]. While hypothetical, it is also plausible that the presence of treatment-sensitive gonorrhoea within an individual can affect the transmissibility and/or susceptibility of treatment-resistant gonorrhoea and vice-versa. To accommodate the range of possibilities, we assume that every individual can be infected with treatment-sensitive and treatment-resistant infection concurrently, and the interactions between infections are represented in the model through adjustments to transmission probabilities. This is achieved through the parameters *α*_*s*_,*ϖ*_*s*_,*ϕ*_*s*_ which are scalar multipliers applied to the transmission probability of treatment-sensitive gonorrhoea should one or both individuals in a sexual partnership already be infected with treatment-resistant gonorrhoea. Likewise, the parameters *α*_*r*_,*ϖ*_*r*_,*ϕ*_*r*_ are the scalar multipliers applied to the transmission probability of treatment-resistant gonorrhoea should one or both partners already be infected with treatment-sensitive gonorrhoea. Fig 1 illustrates this concept and the mechanism by which transmission occurs in the model.

With the appropriate choice of values for the parameters *α*_*s*_,*ϖ*_*s*_,*ϕ*_*s*_,*α*_*r*_,*ϖ*_*r*_,*ϕ*_*r*_, the following scenarios are possible when sexual contact occurs between an individual infected with a treatment-resistant gonorrhoea strain and an individual infected with a treatment-sensitive strain:

I An individual can be co-infected with both strains but there will be no interaction between them, i.e., they will be transmitted independently (*α*_*s*_,*ϖ*_*s*_,*ϕ*_*s*_ and *α*_*r*_,*ϖ*_*r*_,*ϕ*_*r*_ all equal to one)
II Co-infection is not possible:
  - An individual infected with treatment-sensitive gonorrhoea is immune to infection with treatment-resistant gonorrhoea (*α*_*r*_,*ϖ*_*r*_,*ϕ*_*r*_ all equal to zero)
  - An individual infected with treatment-resistant gonorrhoea is immune to infection with treatment-sensitive gonorrhoea (*α*_*s*_,*ϖ*_*s*_,*ϕ*_*s*_ all equal to zero)
III Co-infection is possible but treatment-sensitive gonorrhoea will be converted into treatment-resistant gonorrhoea such that the co-infected individual can only transmit treatment-resistant gonorrhoea (*α*_*s*_,*ϖ*_*s*_,*ϕ*_*s*_ all equal to zero and *α*_*r*_,*ϖ*_*r*_,*ϕ*_*r*_ all equal to one)
IV Co-infection is possible but treatment-resistant gonorrhoea will be converted into treatment-sensitive gonorrhoea such that the co-infected individual can only transmit treatment-sensitive gonorrhoea (*α*_*s*_,*ϖ*_*s*_,*ϕ*_*s*_ all equal to one, and *α*_*r*_,*ϖ*_*r*_,*ϕ*_*r*_ all equal to zero)
  Note that the product of each parameter and the transmission probability (e.g. *α*_*r*_ *β*) must be less than or equal to one
  In the above scenarios, the values for *α*_*s*_,*ϖ*_*s*_,*ϕ*_*s*_ are all either zero or one and similarly for *α*_*r*_,*ϖ*_*r*_,*ϕ*_*r*_. Alternatively the values for these parameters can be varied individually and can take values other than zero or one leading to the following additional scenarios:
V Infection with treatment-sensitive gonorrhoea enhances (*α*_*r*_ > 1, 0 ≤ *α*_*r*_ *β* ≤ 1) or reduces (*α*_*r*_ < 1, 0 ≤ *α*_*r*_ *β* ≤ 1) susceptibility to, or provides immunity against (*α*_*r*_ = 0) infection with treatment-resistant gonorrhoea, or vice versa (with *α*_*e*_ in place of *α*_*r*_).
VI Infection with treatment-sensitive gonorrhoea enhances (*ϖ*_*r*_ > 1, 0 ≤ *ϖ*_*r*_ *β* ≤ 1), reduces (*ϖ*_*r*_ < 1, 0 ≤ *ϖ*_*r*_ *β* ≤ 1) or prevents (*ϖ*_*r*_ = 0) the transmission of treatment-resistant gonorrhoea, or vice versa (with *ϖ*_*s*_ in place of *ϖ*_*r*_)
VII A combination of scenarios V and VI above, governed by parameters *ϕ*_*s*_ and *ϕ*_*r*_ for treatment-resistant and treatment-sensitive strains, respectively.

### Diagnosis and importation treatment-resistant gonorrhoea

We assume that gonorrhoea diagnostic tests have sensitivity of 100% based on the fact that diagnosis in remote settings is predominantly by NAAT [10]. All individuals with a positive diagnosis receive the same standard treatment (i.e. with 5% treatment failure rate for treatment-sensitive gonorrhoea and 100% treatment failure rate for treatment-resistant gonorrhoea) when AMR status is unknown. An alternative effective treatment is administered for infection identified as treatment-resistant, with the failure rate reduced from 100% to the baseline failure rate of 5%.

We also assume that a percentage of samples provided for diagnosis will be suitable for culture-based AMR testing. We investigate three levels: 1) 17% as reported for Western Australia [9]; 2) 22% as reported for the Northern Territory [6]; and 3) 30% of samples from males and 50% of samples from females as reported for Far North Queensland [8]. We assume that the molecular test is able to determine AMR status for all diagnostic samples (whether suitable for culture or not).

At the present time, treatment-resistant gonorrhoea is rarely encountered in remote communities in Australia [20]. Therefore, the emergence of resistance, as implemented in this model, is necessarily hypothetical. We assume treatment-resistant gonorrhoea is introduced into the modelled population periodically in the same way that repeated importation from overseas rather than de novo emergence (through genetic mutation/adaptation) is thought to be the main source of AMR in Australia generally [21]. In order to capture a realistic rise in the prevalence of the hypothetical treatment-resistant gonorrhoea strain, the model is fitted to data on the rise in prevalence of ciprofloxacin resistance in Australia between 2002 and 2008 [22] as this has been well documented. This is achieved through the resistance proportion, which we define as the percentage of infection in the population that is attributable to treatment-resistant gonorrhoea. Calibration is performed by adjusting the six transmission probability adjustment parameters described previously and in Fig 1, using the Nelder-Mead simplex optimisation algorithm [23], such that the sum of the differences between the resistance proportion in the model and the observed data at each time point is minimised.

### Determining the impact of molecular test on AMR surveillance

We assume that gonorrhoea AMR can be determined from gonorrhoea-positive diagnostic samples, either by culture, if the sample is suitable, or by molecular test if not. AMR surveillance is implemented in the model using a simple monitoring scheme whereby an “alert” is triggered when more than 10 of the last 200 (5%) positive diagnoses (for which AMR is determined) are resistant to treatment. The benefit of molecular resistance testing on the timeliness of the alert is assessed using three measures: 1) the resistance proportion when the alert is triggered; 2) the delay between first importation of treatment-resistant gonorrhoea into the population and the triggering of the alert; and 3) the delay between the time when the resistance proportion breaches the 5% threshold and the triggering of the alert.

## Results

Each result consists of the compilation of 1000 simulation runs. Here we divide the findings from the model into 2 sections: 1) the result of model calibration, and 2) the potential impact of molecular test surveillance for gonorrhoea AMR on the timeliness of detection and response.

### Calibration

**Fig 2:**
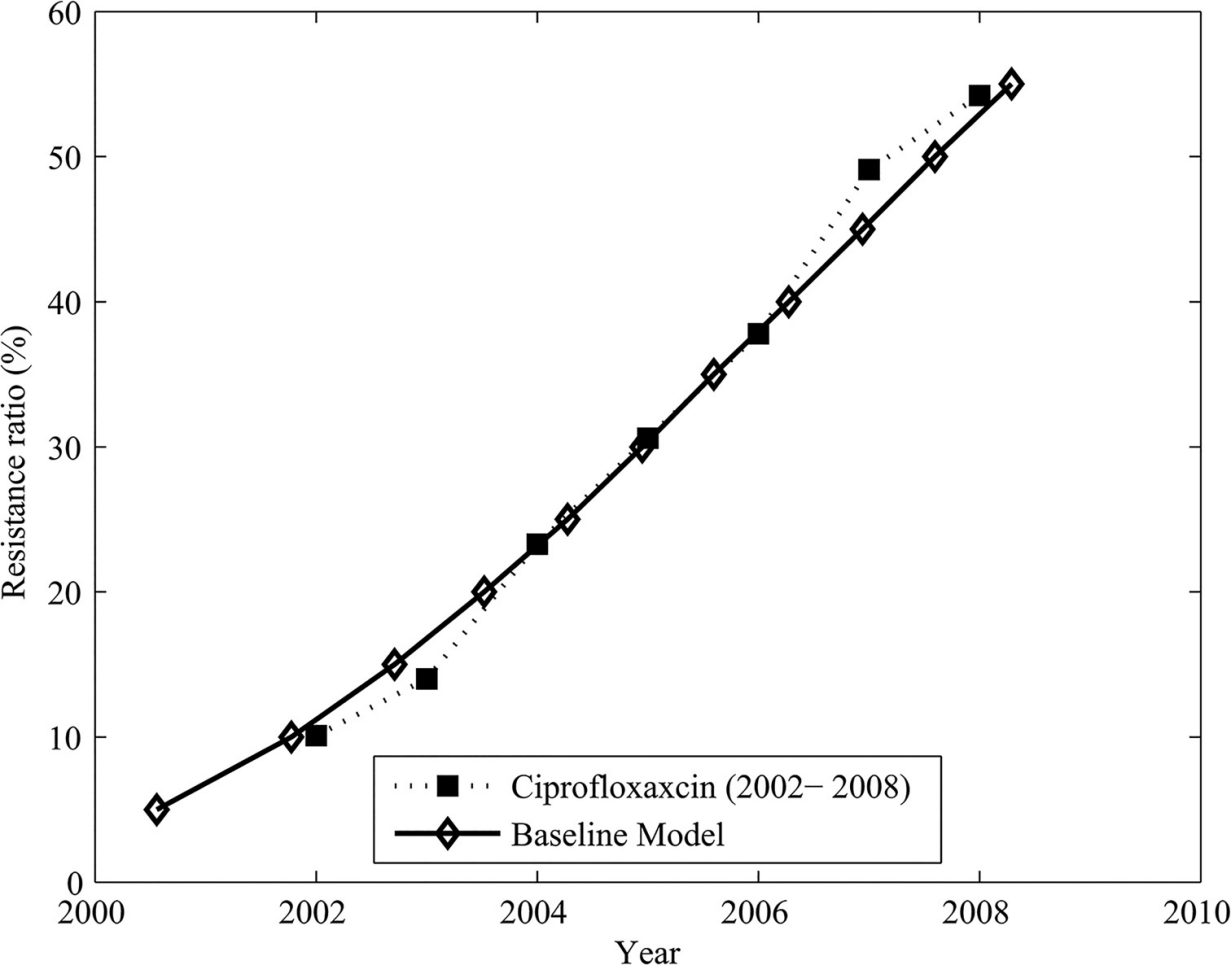
Resistance proportion obtained in the model through calibration to data on ciprofloxacin resistance in Australia. Resistance proportion obtained in the model (solid line with diamond markers) through calibration to data on ciprofloxacin resistance in Australia between 2002 and 2008 (dotted line with square markers, from [22]).

Assuming that on average a single treatment-resistant gonorrhoea infection is imported into the population per year, the modelled resistance proportion increases at a rate similar to the emergence of ciprofloxacin resistance in Australia between 2002 and 2008 if the transmission of treatment-sensitive and treatment-resistant gonorrhoea are independent of each other (with *ϖ*_*s*_ and *ϖ*_*r*_ close to 1), and if the susceptibility to treatment-resistant gonorrhoea is lower when the susceptible individual is already infected with treatment-sensitive gonorrhoea and vice versa (*α*_*s*_ and *ϕ*_*s*_ are close to 0.08, and *α*_*r*_ and *ϕ*_*r*_ are close to 0.15). Table 3 summarises the parameter values obtained from calibration and Fig 2 shows the correspondence between the modelled resistance proportion and ciprofloxacin resistance in Australia between 2002 and 2008.

**Table 3:** Scalar adjustment of transmission probability. Scalar adjustment of transmission probability as derived through the calibration process for the result shown in Fig 2. Explanation of these parameters is given in the Methods and Fig 1.

*α*_*s*_	0.089	*α*_*r*_	0.147
*ϖ*_*s*_	0.999	*ϖ*_*r*_	0.994
*ϕ*_*s*_	0.073	*ϕ*_*r*_	0.149

### Impact of molecular test on AMR surveillance

The resistance proportion at the time the 5% alert is triggered, the delay between first importation and the time of the alert, and the delay between the resistance proportion reaching 5% and the time of the alert are given in Table 4. We compare scenarios where culture alone is available for AMR testing and where both culture and a molecular test are available. When culture alone is available for resistance testing, (upper panel of Table 4), the resistance proportion will already have reached between ~8% and ~18% by the time the alert is triggered, with a delay of ~43–69 months between first importation and the alert being triggered, depending on the percentage of diagnostic samples available for culture. At the highest rate investigated in this study (30% and 50% of diagnostic samples from males and females, respectively suitable for culture-based AMR testing), there is still a 12-month gap between the time the resistance proportion exceeds the 5% threshold and the triggering of the alert.

**Table 4:** Impact of molecular test on AMR surveillance over 1000 simulation runs.

**Culture only**			
**Percentage of diagnoses where AMR can be detected**	**17%** *	**22%** **	**30% in male, 50% in female** ***
Median (IQR) resistance proportion at the time alert is triggered	17.8% (7.9%- 31.3%)	12.5% (5.9%- 23.1%)	8.2% (4.5%- 14.5%)
Median (IQR) time between first importation of treatment-resistant gonorrhoea and time of alert (months)	68.8 (56.7–86.0)	58.9 (47.9–73.0)	43.1 (33.9–56.8)
Median (IQR) time between first instance of resistance proportion exceeding 5% and time of alert (months)	36.5 (19.8–49.2)	26.2 (9.1–39.0)	11.7 (2.6–24.0)
**Culture and molecular test**			
**Percentage of diagnoses where AMR can be detected from culture or molecular test**	**50%**	**75%**	**100%**
Median (IQR) resistance proportion at the time of alert	6.8% (4.1% -10.8%)	6.2% (4.1%- 8.9%)	5.8% (4.0%- 7.9%)
Median (IQR) time between first importation of treatment-resistant gonorrhoea and time of alert (months)	34.3 (25.2–54.9)	30.4 (19.9–58.3)	34.4 (18.1–61.0)
Median (IQR) time between first instance of resistance proportion exceeding 5% and time of alert (months)	6.0 (-1.0–13.2)	4.2 (-0.9–9.1)	3.4 (-1.2–7.4)

IQR = interquartile range. The upper pane represents the situation where a molecular test is not available and AMR can only be detected from culture. The bottom panel represents the situation where AMR can be detected either by culture or molecular test.

*Based on the percentage of isolates available for culture in Western Australia [9]

**Based on the percentage of isolates available for culture in Northern Territory [6]

***Based on the percentage of isolates available for culture in Far North Queensland [8]

The use of molecular testing increases the number of samples for which AMR can be determined and allows the alert to be triggered earlier and at a lower resistance proportion (lower panel of Table 4). For the best-case scenario, where AMR can be determined for all samples, the alert will be triggered at least 8 months earlier than using culture alone and the resistance proportion will have only slightly exceeded the 5% notification threshold. Under this scenario, an alert will be triggered within 3–6 months of the resistance proportion exceeding the 5% threshold, although the negative values in some results suggested that in sometimes the alert is triggered before the threshold is reached under our simplified trigger criterion (10 of the last 200 samples are identified as treatment resistant).

## Discussion

To our knowledge this is the first study to use mathematical modelling to evaluate the potential benefit of a molecular test for gonorrhoea AMR surveillance. We have based the modelled population on remote Australian Indigenous communities because in this setting NAAT is replacing culture for diagnosis and AMR surveillance is thereby being undermined by limited availability of samples for conventional AMR testing. We have implemented a simple monitoring scheme in our model whereby an alert is triggered when 10 of the last 200 samples are identified as treatment resistant. Our findings indicate that if a molecular test for AMR is available, this alert is triggered at least 8 months earlier than when AMR surveillance relies on culture alone. Furthermore, the actual prevalence of treatment-resistant gonorrhoea in the population at the time the alert is triggered is up to 12% lower when a molecular test is available than when culture is the only means of testing for AMR.

Our simple monitoring scheme counts AMR-positive samples from last 200 AMR-identifiable samples used for gonorrhoea diagnosis to determine the timing of the alert. Since the availability of a molecular test will increase the number of samples available for AMR surveillance, those 200 samples will likely be obtained over a shorter period of time. While this reduces the delay in the triggering of the alert, in some cases it the alert may be triggered prematurely (e.g., due to a transient sharp increase in resistance proportion in a small isolated sexual network). In practise, the AMR monitoring scheme is likely to be more complex and will necessarily involve checks and balances designed to minimise premature triggering. Our results highlight the need for these checks and balances to be put in place.

A number of studies employing a range of model structures have investigated the dynamics of emergence and spread of AMR [24], some of which specifically focused on gonorrhoea [25, 26]. However these studies generally assumed de novo development of resistance (e.g. as a result of selective pressure due to antibiotics), while the interactions between strains in co-infected individuals were often simplified or ignored. Such models are not suitable in the context of remote Indigenous communities of Australia as addressed in this study. This is because there is little opportunity for AMR to develop de novo in this population as evidenced by the fact that penicillin is still widely effective. While the prevalence of gonorrhoea in many remote communities is very high, the number of individuals infected is relatively small due to the small population size and unlikely to support the de novo development of antibiotic resistance. For this reason the focus of our study was on importation alone. The model could, however, be extended to also accommodate de novo development of resistance for other settings where this is more likely to occur.

The high prevalence of gonorrhoea infection in this population, however, means that co-infection is likely to occur if there are multiple circulating strains. The model developed for this study allows us to explore a range of scenarios where treatment-resistant gonorrhoea is introduced into the population and co-infection is allowed to occur.

While our results are derived for a remote Indigenous setting in Australia, and may be different for other settings (according to differences in importation rate, sexual behaviour, demographics and health service provision), we believe that the key finding that a molecular test for AMR surveillance will enable more timely detection is robust. The degree to which surveillance will be enhanced by the availability of a molecular test for AMR will be proportional to the extent to which NAAT-based diagnostic testing has replaced culture-based testing. For the setting examined here, we found that the true population resistance proportion at the triggering of the alert is close to the threshold level of 5% if the AMR of all available (gonorrhoea-positive) samples is known. This is an encouraging result as it means that in theory a switch to an alternative effective treatment can be initiated before the number of treatment failures escalates.

Gonorrhoea infections are usually anatomically localised (e.g., urethra, rectum, pharynx) and treatment efficacy may vary by site. For example, cases of ceftriaxone treatment failure for pharyngeal infections have been reported in Australia [27–29] but not for rectal or urethral infection. We have only considered genital infection in the current study as most infections in remote communities are through heterosexual contact [1]. The model can, however, be extended to accommodate transmission involving non-genital sites should the appropriate data become available. We also assume, as explained above, that the emergence of treatment-resistant gonorrhoea in remote Australia is likely to occur through importation rather than in a de novo fashion through selective pressure. While we consider this to be a reasonable assumption [21], the importation frequency required for emergence and persistence in remote communities is unknown. We have assumed an importation rate of one case per year into the modelled population on average for calibration as this generates a reasonable fit to the historical emergence of ciprofloxacin resistance in Australia. A more complete analysis would include the importation frequency as part of the calibration process. However, preliminary simulations have suggested that the importation frequency only affects the rate of emergence at an early stage when the resistance proportion is relatively low (i.e. < 30%). An accurate calibration to importation frequency is not feasible for this model unless more data points are available for the period when the resistance proportion is low.

We assumed a treatment failure rate of 100% for treatment-resistant infections. While in the laboratory environment the treatment failure rate for a specific strain is likely to be less than 100%, the observed treatment failure rate in the population is often unknown at diagnosis (and often remains unknown after treatment due to factors such as non-compliance to treatment, loss to follow-up or reinfection). AMR is identified in the model through strain type rather than treatment failure rate, therefore a lower treatment failure rate will not influence the rate at which treatment-resistant infections are detected. A lower treatment failure rate would reduce the average duration of treatment-resistant infections in the population, but its effect would be compensated for by changes to other factors (e.g. higher transmissibility) through the calibration process.

Our model considers only two generic strains of gonorrhoea–a treatment-sensitive strain and a treatment-resistant strain. In reality a large number of gonococcal strains (each with different sensitivity to particular antibiotic treatment) could be circulating in the population. This model can be expanded to include multiple strains along with strain specific interactions, although the number of parameters required, and the uncertainty associated with each, would lead to much greater overall uncertainty in the predicted outcomes.

Although our results clearly demonstrate the potential benefit of molecular tests for gonorrhoea AMR, we note that such tests should not be considered as a replacement for culture which is required to identify strains of gonorrhoea containing genetic markers that are not recognised by the panel of available molecular tests. Rather, molecular tests for gonorrhoea AMR should be considered as a tool to enhance surveillance in settings where obtaining and maintaining culture is difficult.

In this study, we developed a model to investigate the potential impact of a molecular test on the efficiency of AMR surveillance in remote Indigenous communities with high endemic gonorrhoea prevalence and where the availability of culture is declining. As treatment-resistant gonorrhoea is uncommon in these communities, many aspects of this study are necessarily hypothetical. However, our results indicate that AMR surveillance would be enhanced by the use of a molecular resistance test at diagnosis by enabling more timely detection of resistance and more targeted treatment. Additional AMR surveillance data gained from the use of a molecular test would also enhance our understanding of the dynamics of emergence and spread of treatment-resistant gonorrhoea, facilitate additional refinement of this and other models of AMR, and inform the design of preparedness and prevention strategies for this imminent threat.

## Supporting Information

S1 Technical Appendix Technical Appendix. (DOCX)

## References

[1] The Kirby Institute. Bloodborne viral and sexually transmitted infections in Aboriginal and Torres Strait Islander People: Surveillance and Evaluation Report 2013 Sydney, NSW: The Kirby Institute, the University of New South Wales, 2013.

[2] McDonagh P, Ryder N, McNulty AM, Feedman E. Neisseria gonorrhoeae infection in urban Sydney women: prevalence and predictors. Sexual Health. 2009;6(3):241–4. 10.1071/SH09025 19653962

[3] Guy R, Garton L, Taylor-Thompson D, Silver B, Hengel B, Knox J, et al. The 2010 baseline prevalence study conducted by the STRIVE trial. Australasian Sexual Health Conference; 26–28 Sept; National Convention Centre, Canberra, ACT, Australia2011. p. 139.

[4] Lahra MM, Lo YR, Whiley DM. Gonococcal antimicrobial resistance in the Western Pacific Region. Sex Transm Infect. 2013;89 Suppl 4:iv19–iv23. 10.1136/sextrans-2012-050906 . 24243875

[5] World Health Organization. Global action plan to control the spread and impact of antimicrobial resistance in Neisseria gonorrhoeae Geneva: World Health Organization, 2012.

[6] Northern Territory Department of Health. Sexual Health and Blood Borne Viruses Unit surveillance update. Darwin: 2013.

[7] Trembizki E, Lahra M, Stevens K, Freeman K, Hogan T, Hogg G, et al A national quality assurance survey of Neisseria gonorrhoeae testing. Journal of medical microbiology. 2014;63(Pt 1):45–9. Medline: 10.1099/jmm.0.065094-0 24092762

[8] Fagan PS, Downing SG, McCall BJ, Carroll HJ, Howard TM, Palmer CM. Enhanced surveillance for gonorrhoea in two diverse settings in Queensland in the 2000s: comparative epidemiology and selected management outcomes. Communicable Diseases Intelligence. 2014;37(3). 10.33321/cdi.2013.37.38 24890962

[9] Speers DJ, Fisk RE, Goire N, Mak DB. Non-culture Neisseria gonorrhoeae molecular penicillinase production surveillance demonstrates the long-term success of empirical dual therapy and informs gonorrhoea management guidelines in a highly endemic setting. The Journal of antimicrobial chemotherapy. 2014;69(5):1243–7. Medline: 10.1093/jac/dkt501 24379305

[10] Su J-Y, Pell C. Evidence for a sharp decrease in gonococcal cultures and its implications for the surveillance of antimicrobial sensitivity. In: Markey P, editor. The Northern Territory Disease Control Bulletin2009. p. 11–4.

[11] Goire N, Lahra MM, Chen M, Donovan B, Fairley CK, Guy R, et al Molecular approaches to enhance surveillance of gonococcal antimicrobial resistance. Nat Rev Microbiol. 2014;12(3):223–9. Medline: 10.1038/nrmicro3217 24509781

[12] Goire N, Freeman K, Tapsall JW, Lambert SB, Nissen MD, Sloots TP, et al Enhancing gonococcal antimicrobial resistance surveillance: a real-time PCR assay for detection of penicillinase-producing Neisseria gonorrhoeae by use of noncultured clinical samples. Journal of clinical microbiology. 2011;49(2):513–8. Medline: 10.1128/JCM.02024-10 21159935 PMC3043482

[13] Hui BB, Gray RT, Wilson DP, Ward JS, Smith AMA, Philp DJ, et al Population movement can sustain STI prevalence in remote Australian indigenous communities. BMC Infectious Diseases. 2013;13:188 10.1186/1471-2334-13-188 23618061 PMC3641953

[14] Australian Bureau of Statistics. Estimates of Aboriginal and Torres Strait Islander Australians, June 2011. In: Australian Bureau of Statistics, editor. 2011.

[15] Guy R, Ward JS, Smith KS, Su JY, Huang RL, Tangey A, et al The impact of sexually transmissible infection programs in remote Aboriginal communities in Australia: a systematic review. Sex Health. 2012;9(3):205–12. Epub 16 June 2012. SH11074 [pii] 10.1071/SH11074 . 22697136

[16] Tabrizi S, Twin J, Unemo M, Limnios EA, Guy R. Analytical performance of GeneXpert CT/NG, the first real-time PCR point-of-care assay for the detection of Chlamydia trachomatis and Neisseria gonorrhoeae. 13th IUSTI World Congress; Melbourne Convention and Exhibition Centre, Melbourne, Victoria, Australia2012. p. 72.

[17] Senior K, Helmer J, Chenhall R, Burbank V. 'Young clean and safe?' Young people's perceptions of risk from sexually transmitted infections in regional, rural and remote Australia. Cult Health Sex. 2014;16(4):453–66. Medline: 10.1080/13691058.2014.888096 24592872

[18] Thompson SC, Greville HS, Param R. Beyond policy and planning to practice: getting sexual health on the agenda in Aboriginal communities in Western Australia. Australia and New Zealand Health Policy. 2008;5(3). 10.1186/1743-8462-5-3 PMC2430582 18485244

[19] Lynn F, Hobbs MM, Zenilman JM, Behets FMTF, Van Damme K, Rasamindrakotroka A, et al Genetic typing of the porin protein of Neisseria gonorrhoeae from clinical noncultured samples for strain characterization and identification of mixed gonococcal infections. Journal of clinical microbiology. 2005;43(1):368–75. Medline:. 15634996 10.1128/JCM.43.1.368-375.2005 PMC540152

[20] Lahra M. Annual Report of the Australian Gonococcal Surveillance Programme,2012. Communicable Diseases Intelligence. 2013;37:E233–E9. 24890959 10.33321/cdi.2013.37.35

[21] Tapsall J, Limnios EA, Murphy D. Analysis of trends in antimicrobial resistance in Neisseria gonorrhoeae isolated in Australia, 1997–2006. Journal of Antimicrobial Chemotherapy. 2008;61:150–5. 17986492 10.1093/jac/dkm434

[22] Australian Government Department of Health. Australian Gonococcal Surveillance Programme Annual Reports [cited 2014 13 February]. Available from: http://www.health.gov.au/internet/main/publishing.nsf/Content/cda-pubs-annlrpt-gonoanrep.htm#who.

[23] Lagarias JC, Reeds JA, Wright MH, Wright PE. Convergence properties of the Nelder-Mead simplex method in low dimensions. Siam J Optimiz. 1998;9(1):112–47. Epub 2 December 1998. 10.1137/S1052623496303470 .

[24] Spicknall IH, Foxman B, Marrs CF, Eisenberg JN. A modeling framework for the evolution and spread of antibiotic resistance: literature review and model categorization. Am J Epidemiol. 2013;178(4):508–20. 10.1093/aje/kwt017 23660797 PMC3736756

[25] Pinksky P, Shonkwiler R. A gonorrhea model treating sensitive and resistant strains in a multigroup population. Mathematical Biosciences. 1990;98:103–26. 2134495 10.1016/0025-5564(90)90013-o

[26] Chan CH, McCabe CJ, Fisman DN. Core groups, antimicrobial resistance and rebound in gonorrhoea in North America. Sexually Transmitted Infections. 2012;88(3):200–4. 10.1136/sextrans-2011-050049 22169277

[27] Tapsall J, Read P, Carmody C, Bourne C, Ray S, Limnios A, et al Two cases of failed ceftriaxone treatment in pharyngeal gonorrhoea verified by molecular microbiological methods. J Med Microbiol. 2009;58(Pt 5):683–7. Epub 16 January 2009. 10.1099/jmm.0.007641-0 . 19369534

[28] Chen M, Stevens K, Tideman R, Zaia A, Tomita T, Fairley CK, et al Failure of 500 mg of ceftriaxone to eradicate pharyngeal gonorrhoea, Australia. The Journal of antimicrobial chemotherapy. 2013;68(6):1445–7. 10.1093/jac/dkt017 . 23390207

[29] Read PJ, Limnios EA, McNulty A, Whiley D, Lahra MM. One confirmed and one suspected case of pharyngeal gonorrhoea treatment failure following 500mg ceftriaxone in Sydney, Australia. Sexual health. 2013;10(5):460–2. Medline: 10.1071/SH13077 24028864

[30] Australian Bureau of Statistics. Experimental Estimates and Projections, Aboriginal and Torres Strait Islander Australians, 1991 to 2006 (cat. no. 3238.0). Canberra2009.

[31] Biddle N, Prout S. The geography and demography of Indigenous temporary mobility: an analysis of the 2006 census snapshot. Journal of Population Research. 2009;26:305–26.

[32] Bryant J, Ward J, Worth H, Hull P, Solar S, Bailey S. Safer sex and condom use: a convenience sample of Aboriginal young people in New South Wales. Sexual Health. 2011;8:378–83. 10.1071/SH10138 21851779

[33] Rissel CE, Richters J, Grulich AE, de Visser RO, Smith AMA. Sex in Australia: selected characteristics of regular sexual relationships. Australian and New Zealand Journal of Public Health. 2003;27(2):124–30. 14696702 10.1111/j.1467-842x.2003.tb00799.x

[34] Korenromp EL, Sudaryo MK, De Vlas SJ, Gray RH, Sewankambo NK, Serwadda D, et al What proportion of episodes of gonorrhoea and chlamydia becomes symptomatic? International Journal of STD & AIDS. 2002;13(2):91–101. 11839163 10.1258/0956462021924712

[35] Garnett GP, Mertz KJ, Finelli L, Levine WC, St Louis ME. The transmission dynamics of gonorrhoea: modelling the reported behaviour of infected patients from Newark, New Jersey. Philosophical Transactions: Biological Sciences. 1999;354(1384):787–97. 10365404 10.1098/rstb.1999.0431 PMC1692556

[36] Brunham RC, Garnett GP, Swinton J, Anderson RM. Gonococcal infection and human fertility in sub-Saharan Africa Proceedings of the Royal Society London B: Biological Sciences. 1991;246(1316):173–7. 10.1098/rspb.1991.0141 1663249

[37] Johnson LF, Alkema L, Dorrington RE. A Bayesian approach to uncertainty analysis of sexually transmitted infection models Sexually Transmitted Infections. 2010;86:169–74. 10.1136/sti.2009.037341 19880971 PMC2953461

[38] Platt R, Rice PA, McCormack WM. Risk of acquiring gonorrhea and prevalence of abnormal adnexal findings among women recently exposed to gonorrhea. Journal of the American Medical Association. 1983;250(23):3205–9. 6417362

[39] Potterat JJ, Dukes RL, Rothenberg R. Disease transmission by heterosexual men with gonorrhea: an empiric estimate. Sexually Transmitted Diseases. 1987;14(2):107–10. 3616851 10.1097/00007435-198704000-00010

[40] Holmes KK, Johnson DW, Trostle HJ. An estimate of the risk of men acquiring gonorrhea by sexual contact with infected females. American Journal of Epidemiology. 1970;91(2):170–4. 5416250 10.1093/oxfordjournals.aje.a121125

