# Supplementary material for: Exploring the Benefits of Molecular Testing for Gonorrhoea Antibiotic Resistance Surveillance in Remote Settings
Source: PLoS One. 2015 Jul 16;10(7):e0133202. doi: 10.1371/journal.pone.0133202 (PMC4504484; doi:10.1371/journal.pone.0133202)
Supplement: S1 Technical Appendix — (DOCX) [file pone.0133202.s001.docx]

# S1 Technical appendix

## Overview

The modelled population consists of 5000 heterosexual individuals spanning 5 hypothetical locations. The first location comprises the home location for 4000 individuals, while the remaining 4 locations are home to 250 individuals each. Individuals can move to other locations throughout the simulation, but their home location does not change once it has been set.

The location, infection and partnership status of all individuals in the model is tracked daily over the course of 50 years. For simplicity, in this document as well as in the main text, a month refers to a period of 30 days and a year is a period of 12 months or 360 days.

All individuals in the model are assigned an age between 15 and 35 years, with the gender-age distribution maintained as described in the Australian Bureau of Statistics census estimate for remote Indigenous communities in 2011 [[1](#_ENREF_1)]. This is achieved through adjustment to gender- and age-specific mortality/removal rates. For example, assume there are 2500 males in total, of which 27% (or 675) is currently aged 20 to 25 years. If on one particular day, 5 individuals have their 26th birthday, while 10 individuals have their 20th birthday, the number of individual aged 20-25 will be 675-5+10 = 680). In this scenario 5 individuals (out of 680) will be randomly chosen from the population and removed, to be replaced by a new individual aged 15.

Each simulation runs begin with a burn-in period of 100 years, such that the prevalence of treatment sensitive gonorrhoea is maintained at 7-8% for at least the last 50 years. Treatment resistant gonorrhoea is then introduced into the population periodically, and the prevalence of both treatment-sensitive and treatment-resistant gonorrhoea is tracked for a further 50 years.

## Population mobility

As described in a previous modelling study [[2](#_ENREF_2)], the model accounts for temporary mobility, which entails the movement of individuals away from their home location to a new location. Individuals stay at the new location for a pre-determined period before returning to their home location. Cyclic mobility (moving from one non-home location to another) is not considered in this study due to a lack of data.

The number of individuals to move at each time step is determined based on the findings reported in the census study by Biddle and Prout [[3](#_ENREF_3)]. For example, let us assume that at time step *t* there were 500 males aged 15 to 20 years within the population. According to the Table 1 in the main paper, 50 (or 10%) of these individuals should be away from their home location at this time. Let us also assume that there were 45 males aged 15 to 20 years away from their home location at time step *t*. This means that at the next time step (i.e. *t* +1), 5 males aged 15 to 20 will move away, bringing the number of males in this age-group who are away from home to 50 at time step *t* +1. During a simulation run however, the exact numbers of non-residents (and hence number of movements) at a given time step can vary due to factors such as non-residents returning home and the aging of the population.

We assume that individuals moving away from home will select their new destination based on a random selection process weighted by the population size of each location (including both residents and non-residents). For example, an individual will be twice as likely to move to a location with 500 individuals than to one with 250 individuals.

We assume that the duration of time an individual will be away from home will be in the range 2 – 14 days. Five percent of the non-resident population can seek additional partners at any given time (see section “Partnership formation and sexual behaviour”). Both of these parameters are determined through calibration of the model to prevalence data [[2](#_ENREF_2)].

## Partnership formation and sexual behaviour

At each time step sexual partnerships can form between individuals who are currently in the same location. Sexual acts and gonorrhoea transmission can occur within partnerships only when both partners are contemporaneously in the same location. Partnerships are maintained until dissolution even if one or both partners move to different locations, and sexual activity can resume when they are again located in the same location.

Every individual in the model can have one regular and one casual partner concurrently, although the majority will only have one partner at a time. Regular partnerships can only be formed between individuals who share the same home location, while casual partnerships can be formed as long as both partners are at the same location at the formation of the partnership.

The frequency at which new partners are sought, and the length and type of partnership (regular or casual) formed for each individual are assigned on the basis of results from Bryant et al [[4](#_ENREF_4)]. The implementation of this is as follows. The maximum number of partners an individual can have before age 30 is determined by random draw from a Poisson distribution with a mean value of 5. During simulation, approximately 55% of the population will have at least one regular partner within the last six-month period. Approximately 72% of the population will have at least one casual partner in the last six-month period, and approximately 60% will not seek new partners unless there has been a gap of at least 6 months since the commencement of their last partnership.

The length of a regular partnership is determined by random draw from an exponential distribution, with mean 2 years. The length of casual partnerships is not explicitly specified in the model, but casual partnerships are terminated as soon as a new partner is sought. The frequency at which individuals seek partners is determined by their partner acquisition rate. For example, a male assigned a partner acquisition rate of 3 partners per 6 months who has already had 1 casual partner in the last 6 months will seek his next casual partner after 6 x 30 / (3 -1) = 90 days.

To our knowledge nothing has been published on the link between an individual's sexual behaviour and their mobility in the context of remote Indigenous communities in Australia. We therefore assume that the number of partners an individual can have (before age 30) will include all partners at home as well as away. We further assume that the majority of individuals do not seek new partners if they are already within a partnership. However a small percentage of the population (5% in this study) is allowed to seek additional partners while away from home and the partner acquisition rate for these individuals will be calculated based on the number of partners they have had since their arrival at the new location rather than the usual six-month window. Let us take again for example a male with an assigned partner acquisition rate of 3 per 6 months and 1 casual partner in last 6 months. Upon travel to a new non-home location, he may be able to seek and form a new casual partner immediately of the duration of which will be 6 x 30 / (3 - 0) = 60 days. In contrast, if he is unable to seek additional partners upon travel, he will not seek a new casual partner until his current casual partnership expires, and the length of any new partnership will be 6 x 30 / (3 - 1) = 90 days.

The frequency of sex within both casual and regular partnerships is assumed to be similar to that of the general Australian population at 3 times per week [[5](#_ENREF_5)], while condom usage for regular and casual partners are as reported by Bryant et al [[4](#_ENREF_4)]. We assume condoms are 100% effective at preventing transmission of gonorrhoea.

## Natural history of gonorrhoea and gonorrhoea resistance

Transmission of gonorrhoea can occur within sexual partnerships involving infected individuals. The probability of Transmission is determined by the frequency of sex and the per-act probability of transmission. The progression and resolution of infection for an infected individual is tracked over time.

Individuals enter the sexually active population susceptible to infection (i.e., uninfected). Susceptible individuals can acquire infection through sexual contact whereupon they initially enter the ‘Exposed’ state and are not yet infectious. Following this period of latency, exposed individuals then become ‘Infectious’ either asymptomatically or symptomatically and return to the susceptible state either naturally or as a result of receiving treatment.

The transmissibility and natural history of gonorrhoea, including gender-specific durations of infection and immunity and per-act transmission probability have not been fully elucidated and a wide range of values for natural history parameters are quoted in the published literature. The values assigned for gonorrhoea natural history parameter in this study are listed in Table 2 of the main paper.

We assume that untreated gonorrhoea infection will resolve spontaneously within 185 days on average. The duration of infection is thus drawn from a gamma distribution with a mean of 185 days and standard deviation of 5 weeks, based on the modelling study of Johnson et al. [[6](#_ENREF_6)] The duration of infection is shorter in those who receive treatment. An infection will be treated if it is identified through: a) annual screening; b) recognition of symptoms; or c) partner notification.

We assume that treatment-sensitive and treatment-resistant strains of gonorrhoea have identical natural history, and that infection with the respective strains differs only with respect to the likelihood of treatment failure and possible variations in transmission probability and/or susceptibility to infection in the case of co-infection due to strain interaction. The nature of intra- and inter-host interactions between treatment-sensitive and treatment-resistant strains of gonorrhoea are largely unknown. While it is known that treatment-sensitive strains of gonorrhoea have the ability to acquire resistance to treatment through mutation or horizontal transfer [[7](#_ENREF_7)], the dynamics of these events is not well understood necessitating a hypothetical approach to their implementation in our model. The model makes use of 6 parameters to describe a range of possibilities, and the calibration process (against historical data on the emergence of ciprofloxacin resistance in Australia) is used to estimate the possible value for each one of these. Figure 1 of the main text illustrates our implementation, and a description of possible scenarios that this implementation accommodates is given in the text.

**Impact of importation frequency on resistance proportion**

The study assumed an importation rate of one case per year as the starting point for the calibration against the historical emergence of ciprofloxacin resistance in Australia. Given treatment-resistant gonorrhoea is yet to emerge in most remote communities, this importation rate is necessarily hypothetical.

An increase in importation rate will lead to treatment-resistant gonorrhoea entering the community more frequently, and consequently will result in an increase in the rate at which the resistance proportion increases. In our model however, this increase will be compensated for by a reduced transmission rate for the treatment-resistant strain that will occur as a result of the calibration process.

If we assume the transmission rate for the treatment-resistant strain is fixed, then changes to importation frequency will influence the rate at which treatment-resistant gonorrhoea emerges. As Figure A1 illustrates, a decrease in the importation frequency results in an increase in the rate of AMR emergence, hence an increase in the time required for the resistance proportion to reach 5%.


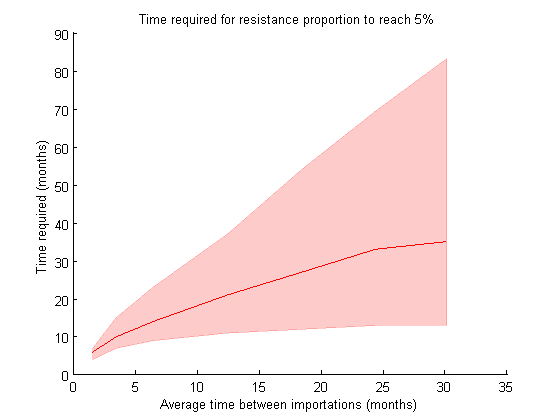


Figure A1: The time required for the resistance proportion to reach 5% (since first case of importation) as a function of the importation frequency (with the transmission probabilities for the treatment-resistant strain fixed to the value defined in the manuscript). The solid line denotes the median time required and the shaded region denotes the interquartile range.

# References

# 1. Australian Bureau of Statistics (2011) Estimates of Aboriginal and Torres Strait Islander Australians, June 2011. In: Australian Bureau of Statistics, editor.

# 2. Hui BB, Gray RT, Wilson DP, Ward JS, Smith AMA, et al. (2013) Population movement can sustain STI prevalence in remote Australian indigenous communities. BMC Infectious Diseases 13: 188.

# 3. Biddle N, Prout S (2009) The geography and demography of Indigenous temporary mobility: an analysis of the 2006 census snapshot. Journal of Population Research 26: 305-326.

# 4. Bryant J, Ward J, Worth H, Hull P, Solar S, et al. (2011) Safer sex and condom use: a convenience sample of Aboriginal young people in New South Wales. Sexual Health 8: 378-383.

# 5. Rissel CE, Richters J, Grulich AE, de Visser RO, Smith AMA (2003) Sex in Australia: selected characteristics of regular sexual relationships. Australian and New Zealand Journal of Public Health 27: 124-130.

# 6. Johnson LF, Alkema L, Dorrington RE (2010) A Bayesian approach to uncertainty analysis of sexually transmitted infection models Sexually Transmitted Infections 86: 169-174.

# 7. Ameyama S, Onodera S, Takahata M, Minami S, Maki N, et al. (2002) Mosaic-like structure of penicillin-binding protein 2 gene (penA) in clinical isolates of Neisseria gonorrhoeae with reduced susceptibility to cefixime. Antimicrobial Agents and Chemotherapy 46: 3744-3749.
